# Supplementary material for: Self-Management Support for Cancer Survivors: A Descriptive Evaluation of the Symptom Navi Training from the Perspective of Health Care Professionals
Source: Curr Oncol. 2025 Jun 2;32(6):326. doi: 10.3390/curroncol32060326 (PMC12191559; doi:10.3390/curroncol32060326)
Supplement: Supplementary file 1 [file curroncol-32-00326-s001.zip › curroncol-3587052-supplementary.pdf]

Supplementary file to “Self-management support for cancer survivors: a descriptive evaluation of the Symptom Navi training from the perspective of health care professionals”

Explorative Factor Analysis of the Work-SoC scale

The EFA revealed a three factor-structure *comprehensibility*, *meaningfulness* and *manageability* each of it consisting of three variables (table S1). These factors explain 53% of the variance in the data, with each factor contributing significantly. No confirmatory factor analysis was conducted. This study was explicitly designed for exploratory scale clarification, so we limited the psychometric work to an EFA. Our reporting therefore focuses on the established EFA diagnostics - eigenvalues, scree and parallel analyses, item communalities and the rotated loading pattern. The eigenvalues for the three extracted factors were 1.69, 1.62, and 1.42, respectively.

Table S1. Factor loading for Work-SoC variables.

Items [1]	Comprehensibility	Meaningfulness	Manageability
- structured	**0.66**	0.12	0.36
-clear	**0.72**	0.25	0.36
-manageable	**0.53**	0.33	0.2
- rewarding	0.19	**0.8**	0.02
- significant	0.14	**0.67**	0.08
- meaningful	0.09	**0.57**	0.1
- easy to influence	0.18	0.07	**0.67**
- controllable	0.48	0.13	**0.6**
- predictable	0.36	0.08	**0.54**

[1] Work-SoC items are listed in order to factor loading results.

The factor structure aligns well with the theoretical expectations and demonstrates good model fit, indicated by favorable fit indices such as a low RMSR indicating minimal discrepancies between the model’s predicted correlations and observed data; and high TLI reflecting that the proposed model explains the data substantially better than a simpler baseline model (figure S1). The RMSR was 0.02 and the Tucker-Lewis Index (TLI) was 1.004, indicating good model fit.

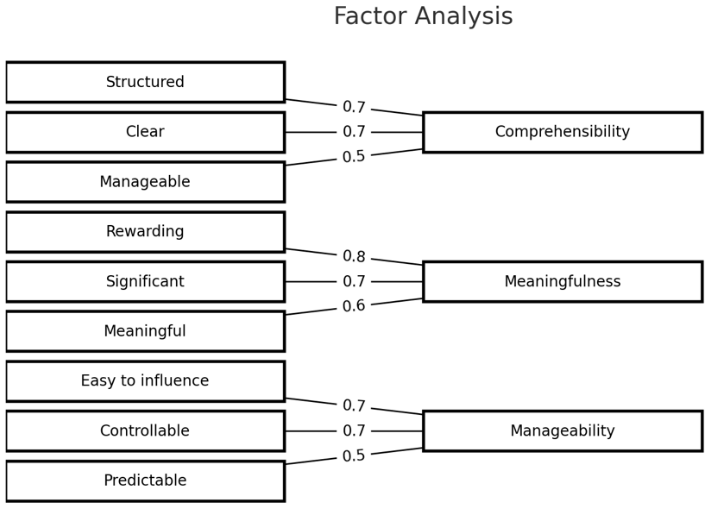

Figure S1. Factor Analysis of the WorkSoC scale. The numbers in this Figure are standardized factor loadings obtained from the Principal Axis Factoring solution after Varimax rotation.
